# Comprehensive Analysis of Cucumber Gibberellin Oxidase Family Genes and Functional Characterization of *CsGA20ox1* in Root Development in *Arabidopsis*

**DOI:** 10.3390/ijms19103135

**Published:** 2018-10-12

**Authors:** Hong Sun, Baoya Pang, Jun Yan, Ting Wang, Lina Wang, Chunhua Chen, Qiang Li, Zhonghai Ren

**Affiliations:** 1 State Key Laboratory of Crop Biology, Shandong Collaborative Innovation Center of Fruit & Vegetable Quality and Efficient Production, Key Laboratory of Biology and Genetic Improvement of Horticultural Crops in Huang-Huai Region, Ministry of Agriculture, College of Horticultural Science and Engineering, Shandong Agricultural University, Tai’an 271018, China; sunh95@163.com (H.S.); pbys521104@163.com (B.P.); 19906456735@163.com (T.W.); lnwang007@163.com (L.W.); dmxyyc@163.com (C.C.); 2 College of Information Science and Engineering, Shandong Agricultural University, Tai’an 271018, China; xinsinian2006@163.com

**Keywords:** cucumber, gibberellin, GAox, expression patterns, *CsGA20ox1*, root development

## Abstract

Cucumber (*Cucumis sativus* L.) is an important vegetable crop worldwide and gibberellins (GAs) play important roles in the regulation of cucumber developmental and growth processes. GA oxidases (GAoxs), which are encoded by different gene subfamilies, are particularly important in regulating bioactive GA levels by catalyzing the later steps in the biosynthetic pathway. Although GAoxs are critical enzymes in GA synthesis pathway, little is known about *GAox* genes in cucumber, in particular about their evolutionary relationships, expression profiles and biological function. In this study, we identified 17 *GAox* genes in cucumber genome and classified them into five subfamilies based on a phylogenetic tree, gene structures, and conserved motifs. Synteny analysis indicated that the tandem duplication or segmental duplication events played a minor role in the expansion of cucumber *GA2ox*, *GA3ox* and *GA7ox* gene families. Comparative syntenic analysis combined with phylogenetic analysis provided deep insight into the phylogenetic relationships of *CsGAox* genes and suggested that protein homology CsGAox are closer to AtGAox than OsGAox. In addition, candidate transcription factors BBR/BPC (BARLEY B RECOMBINANT/BASIC PENTACYSTEINE) and GRAS (GIBBERELLIC ACID-INSENSITIVE, REPRESSOR of GAI, and SCARECROW) which may directly bind promoters of *CsGAox* genes were predicted. Expression profiles derived from transcriptome data indicated that some *CsGAox* genes, especially *CsGA20ox1*, are highly expressed in seedling roots and were down-regulated under GA_3_ treatment. Ectopic over-expression of *CsGA20ox1* in *Arabidopsis* significantly increased primary root length and lateral root number. Taken together, comprehensive analysis of *CsGAoxs* would provide a basis for understanding the evolution and function of the *CsGAox* family.

## 1. Introduction

Gibberellins (GAs), a large group of tetracyclic diterpenes, control diverse aspects of plant growth and development throughout the life cycle of plants, including seed germination [1], stem elongation [2,3], leaf expansion [4], alteration of sex expression [5,6,7], flower and root development [8,9,10] and fruit set and development [11,12]. Although 136 naturally occurring GAs have been discovered [13], most of these molecules have been identified as non-bioactive GAs in plants, and these act as precursors for the bioactive forms or are de-activated metabolites [14]. GA1 and GA4 are the major bioactive GAs with relatively high abundance in various plant species, while GA3 and GA7 are less abundant [14,15].

The gibberellin biosynthetic pathway has been extensively studied in plants [14,15,16]. The biosynthesis and deactivation of GA mainly involves three stages of reactions according to their subcellular compartmentalization and the enzymes involved. The first stage, which is catalyzed by soluble enzymes and occurs in plastids, leads to the synthesis of the tetracyclic hydrocarbon and entkaurene. In the second stage, GA12 and GA53, which constitute the general GA precursors, are synthesized from entkaurene, and further catalyzed by cytochrome P-450 mono-oxygenases at the endoplasmic reticulum. The final stage of the pathway, which has been identified as primarily responsible for the regulation of bioactive GA synthesis, is catalyzed by two GA oxidases (GAoxs) known as GA 20-oxidase (GA20ox) and GA 3-oxidase (GA3ox) in the cytosol of the cell [17,18]. In the pathways and regulation of GA degradation, bioactive GAs or their immediate precursors are inactivated by the third family of GAoxs, the GA 2-oxidases (GA2oxs), including C19-GA2oxs and C20-GA2oxs [14,19].

In fact, GAoxs that catalyze the late steps in the pathway are each encoded by small gene families [20]. The *GAoxs* gene family members and their biological functions have been studied in a variety of plant species [11,20,21]. In *Arabidopsis*, sixteen *GAoxs* genes (five *GA20oxs*, seven *GA2oxs* and four *GA3oxs*) have been identified [20,22]. In rice, 21 *GAoxs* genes have been recognized (eight *GA20oxs*, eleven *GA2oxs* and two *GA3oxs*) [20,23]. The identification of *GAoxs* has provided a clearer view of the mechanism by which a large variety of GAs are produced in plants and manipulating the expression of *GAoxs* enables regulation of the levels of endogenous active GAs in some plant species. For example, overexpression of *GA20ox* and *GA3ox* showed GA-overproduction phenotype in different plants [22,24,25,26,27,28]. Overexpression of *GA2ox* genes in plants causes deficiency in endogenous GAs, leading to dwarf plants [11,23,29,30,31]. 

Cucumber (*Cucumis sativus* L.) is an economically important crop cultivated worldwide [32] and has been used as a model plant for studying hormonal regulation of reproductive development [33,34]. Furthermore, GA was demonstrated to play an important role in flower and fruit development in cucumber [7,18,33,35]. In cucumber, the final part of the GA biosynthetic pathway is catalyzed by four subfamilies of GAoxs: GA 7-oxidases (GA7oxs), GA20oxs, GA3oxs and GA2oxs [36]. Biologically inactive precursor GA12-aldehyde is converted to bioactive GA4 by GA7oxs, GA20oxs and GA3oxs. The GA4 is further oxidised by GA2ox to form biologically inactive GA34 [18,36]. It is worthy to note that GA7ox, which oxidizes GA12-aldehyde to GA12 and possesses mono-oxygenase 7-oxidase activity has been reported in cucumber and pumpkin, but it has not been found in other species [37,38]. 

Although some GAoxs involved in the GA pathway of cucumber have been identified previously [36], what is known about them in cucumber is mainly limited to automatic gene prediction, annotation and catalytic functions, but systematic evolutionary analysis, tissue specificity, timing of expression under GA treatment and their biological function have been neither verified nor explored. Therefore, in this study, we identified 17 *GAox* genes in cucumber and the comprehensive analysis including the gene structure and motif compositions, synteny analysis and gene duplications, phylogenetic relationship, promoter conserved motifs and candidate transcription factors which might directly bind the promoter of CsGAoxs, were further investigated. In addition, based on RNA-seq data the *CsGAox* genes expression profiles in different cucumber tissues and in the roots of cucumber seedlings under GA and uniconazole (Uni, GA biosynthesis inhibitor of ent-Kaurene oxidase) treatments were determined. Furthermore, stable transgenic *Arabidopsis* with overexpression of *CsGA20ox1* yielded longer primary roots and more lateral roots than wildtype, indicating the *CsGA20ox1* is involved in root development in *Arabidopsis*. 

## 2. Results

### 2.1. Genome-Wide Identification and Analysis of GAox Genes in Cucumber

To identify *GAox* family genes in cucumber genome, the 16 *Arabidopsis* GAox proteins and the consensus protein sequences of 2OG-FeII_Oxy (PF03171) and DIOX_N (PF14226) were employed as a query to search against the cucumber genome database using the BlastP program. After removing redundant proteins, a total of 53 putative candidate proteins were obtained. A phylogenetic tree was constructed using the 53 identified proteins from cucumber and 16 GAox proteins of *Arabidopsis*. Then, a total of 21 proteins in the cucumber genome were identified as possible members of the *CsGAox* family (Figure S1). To confirm the presence of the 2OG-FeII_Oxy and DIOX_N domain in putative cucumber GAox proteins, the amino acid sequences of all 21 proteins were searched by Pfam (available online: http://pfam.janelia.org/) and SMART (available online: http://smart.embl-heidelberg.de/). Four proteins (Csa2G379320.1, Csa3G081890.1, Csa3G535100.1, Csa7G435480.1) were excluded as they do not possess a DIOX_N domain, indicating that the 17 proteins are cucumber GAox family members (Figure S1). 

### 2.2. Phylogenetic Analysis of the CsGAox Gene Family

**Figure 1:**
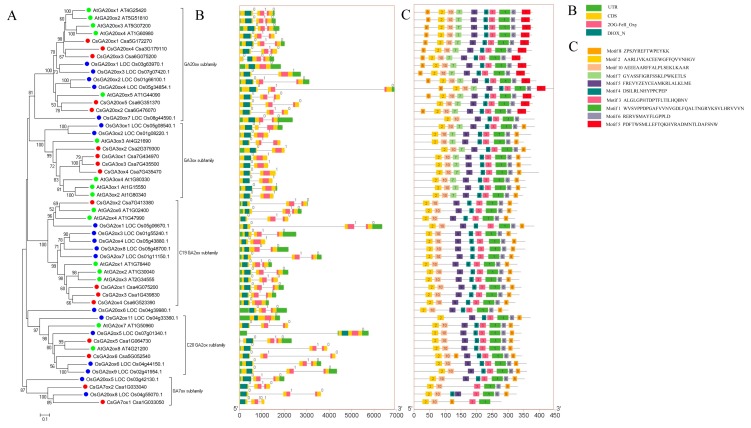
Phylogenetic relationships, gene structure and conserved protein motifs in *GAox* genes from cucumber. (**A**) The phylogenetic tree was constructed based on the full-length protein sequences of 17 CsGAox, 16 AtGAox and 19 OsGAox proteins using MEGA 7.0 software. Cucumber, *Arabidopsis* and rice GAoxs were labeled by red, green and blue dots; (**B**) Exon-intron structure of *CsGAox* genes. Black lines indicate introns. The 2OG-FeII_Oxy and DIOX_N domain is highlighted by red and dark green boxes, respectively. The number indicates the phases of corresponding introns; (**C**) The motif composition of CsGAox proteins. The motifs, numbers 1–10, are displayed in different colored boxes. The sequence logos and *E* values for each motif are given in Figure S3.

To better understand the evolutionary relationships of cucumber *GAoxs*, an unrooted neighbor-joining (NJ) phylogenetic tree using bootstrap analysis (1000 replicates) was built from alignments of the GAox complete protein sequences from 17 CsGAox, 16 AtGAox and 19 OsGAox (Figure 1). The resulting tree generated five distinct subgroups. Among the 17 CsGAox proteins, 5 belong to GA20ox subfamily, 4 to GA3ox subfamily, 6 to GA2ox subfamily and 2 to GA7ox subfamily. The phylogenetic analysis also confirmed the presence of two discrete subgroups of putative GA2ox proteins that belong to either C19 or C20 GA classes. Two cucumber GA2ox members (CsGA2ox5 and 6) belong to C20 subgroup with 2 AtGA2oxs (AtGA2ox7 and 8) and 4 OsGA2oxs (OsGA2ox5, 6, 9 and 11) and 4 cucumber GA2ox members (CsGA2ox1, 2, 3 and 4) to C19 subgroup with 5 AtGA2oxs (AtGA2ox1, 2, 3, 4 and 6) and 7 OsGA2oxs (OsGA2ox1, 3, 4, 7 and 8).

Phylogenetic analysis also revealed that there was not equal representation of cucumber, *Arabidopsis* and rice GAox proteins within the given subgroups. Three subfamilies (GA20ox, GA3ox and GA2ox) were shared in all the 3 species. Among them, GA3ox subfamily included 4 CsGA3ox, 4 AtGA3ox and 2 OsGA3ox proteins, which suggested that this is an expanded subgroup in cucumber and *Arabidopsis* compared with that of rice. GA7ox, that oxidizes GA12-aldehyde to GA12 and possess mono-oxygenase 7-oxidase activity, was reported in pumpkin and cucumber but has not been found in other species [16,36,37]. Strikingly, OsGA20ox5 and 8 were clustered into GA7ox subfamily with CsGA7ox1 and 2, indicating OsGA20ox5 and 8 shared high similarities with CsGA7ox1 and 2. OsGA20ox6 did not fit well into any subfamily, which was also indicated previously [20]. To confirm this result, a multiple sequence alignment (MSA) analysis was also performed with 5 CsGA20ox proteins, 5 AtGA20ox proteins, 8 OsGA20ox protein and 2 CsGA7ox proteins. A GA20ox conserved sequence LPWKET, which was identified previously [21,39], was identified in all of the GA20ox proteins from cucumber and *Arabidopsis*, but not in OsGA20ox5, 6, 7 and 8, which is consistent with the result of phylogenetic analysis (Figure 1 and Figure S2). 

### 2.3. Gene Structure and Conserved Motif Analysis of CsGAox Gene Family

To support the phylogenetic analysis, gene structure analysis of *GAox* family members from cucumber, *Arabidopsis* and rice was performed. As shown in Figure 1, the number of exons in *CsGAox*, *AtGAox* and *OsGAox* genes was conserved, ranging from 1 to 3 exons. We found that the gene structures of putative *GAox* members in the same group were highly conserved in all 3 species (Figure 1). The number of introns contained in their 2OG-FeII_Oxy and DIOX_N domains was also determined (Figure 1B). There was no intron in DIOX_N domain of all 52 *GAox* genes. There was 1 intron in the 2OG-FeII_Oxy domain in all of *GA20ox* genes from cucumber, *Arabidopsis* and rice, except *OsGA20ox1* and *OsGA20ox3*. Eight out of 10 *GA3ox* genes from the 3 species have no intron in the 2OG-FeII_Oxy domain, while *OsGA3ox1* and *AtGA3ox3* have 1 intron in the 2OG-FeII_Oxy domain. In *GA2ox* subfamily, only *CsGA2ox4, OsGA2ox4*, *OsGA2ox5* and *OsGA2ox11* have no intron in the 2OG-FeII_Oxy domain. The 2OG-FeII_Oxy domain in all of the 4 *GA7ox* subfamily members was separated by 1 intron. Intron phases with respect to codons were also investigated. Intron phase 0, 1 and 2 indicates splicing occurred after the first nucleotide, the second nucleotide and the third nucleotide of the codon, respectively. All of the first intron is a phase 0 intron and the second intron is generally a phase 1 intron. This suggested that the splicing phase was also highly conserved during the evolution of GAox genes in cucumber, *Arabidopsis* and rice.

To investigate the motifs that are shared among related proteins within the same subfamily, 10 distinct motifs were identified by the MEME motif search tool (Figure 1C). Motif 2 and 10, which are representative DIOX_N domain, and motif 1 and 3, which are representative 2OG-FeII_Oxy domain, were identified in all GAox proteins. Motif 4, 6, 8 and 9 were identified in most of the GAox proteins. Interestingly, motif 8 was generally located in N-terminal of GA20ox protein, but in C-terminal of GA3ox, GA2ox and GA7ox proteins. Some of the specific motifs were absent in specific subfamilies. For example, motif 7 was absent in all the members of GA2ox and GA7ox subfamily. Motif 5 was only identified in proteins of GA20ox subfamily. Therefore, the functions of these motifs in relation to the functions of these proteins need to be investigated further. 

In summary, the results of gene structure and conserved motif analyses additionally support the results of phylogenetic analysis, illustrating that the evolution of each subfamily was well conserved in three different species.

### 2.4. Synteny Analysis of GAox Genes in Cucumber, Arabidpsis and Rice

**Figure 2:**
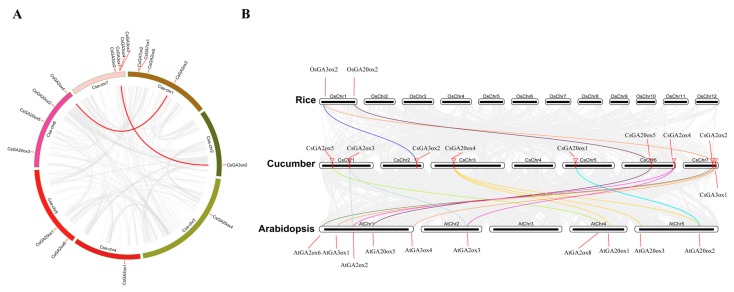
Gene duplication and synteny analysis of *CsGAox* genes. (**A**) Schematic representations for the chromosomal distribution and interchromosomal relationships of *CsGAox* genes. Gray lines indicate all synteny blocks in the cucumber genome, and the red lines indicate segmental duplicated *CsGAox* gene pairs; (**B**) Synteny analysis of *GAox* genes between cucumber and *Arabidopsis* and rice. Gray lines in the background indicate the collinear blocks within cucumber and *Arabidopsis* and rice genomes, while the red lines highlight the syntenic *GAox* gene pairs. Different gene pairs were highlighted by lines with different colors.

Gene duplication, including segmental and tandem duplications, is one of the primary driving forces in the evolution of genomes [40]. Duplication of genes can occur as transfer of the duplicated segment to a site contiguous to the original one (tandem duplication), or it can involve the duplication of large stretches of DNA containing many genes (segmental duplication) [41]. To reveal the duplication of *CsGAox* genes, the syntenic regions were analyzed by MCscanX software. As shown in Table S1, a total of 3789 tandem duplication gene pairs and 177 segmental duplication blocks were identified in the cucumber genome, respectively. Only 1 tandem duplication gene pair was obtained in *CsGAox* family (*CsGA7ox1* and *CsGA7ox2*). In addition, 2 segmental duplication events with 2 *GAox* gene pairs (*CsGA2ox3* and *CsGA2ox4*; *CsGA3ox1* and *CsGA3ox2*) were also identified in cucumber (Figure 2A, Table S1). 

To further expound the phylogenetic mechanisms of cucumber *GAox* family, comparative syntenic maps of cucumber associated with *Arabidopsis* and rice was constructed, respectively (Figure 2B). Three (*CsGA3ox1*, *CsGA3ox2* and *CsGA20ox5*) and eight (*CsGA2ox2*, *CsGA2ox3*, *CsGA2ox4*, *CsGA2ox5*, *CsGA3ox1*, *CsGA20ox1*, *CsGA20ox4* and *CsGA20ox5*) *CsGAox* genes showed syntenic relationship with those in rice and *Arabidopsis*, respectively. Interestingly, some *CsGAox* genes were found to be associated with at least two syntenic gene pairs between cucumber and *Arabidopsis*, such as *CsGA2ox3*, *CsGA2ox4*, *CsGA3ox1* and *CsGA20ox4*, indicating that these genes may have played an important role of *GAox* gene family during evolution. Additionally, some collinear pairs (with *CsGA3ox1* and *CsGA20ox5*) were identified between cucumber and both *Arabidopsis* and rice, indicating that these orthologous pairs may already exist before the ancestral divergence. 

### 2.5. Conserved Motif and Transcription Factor Binding Site Analysis in the Promoter of CsGAoxs

**Figure 3:**
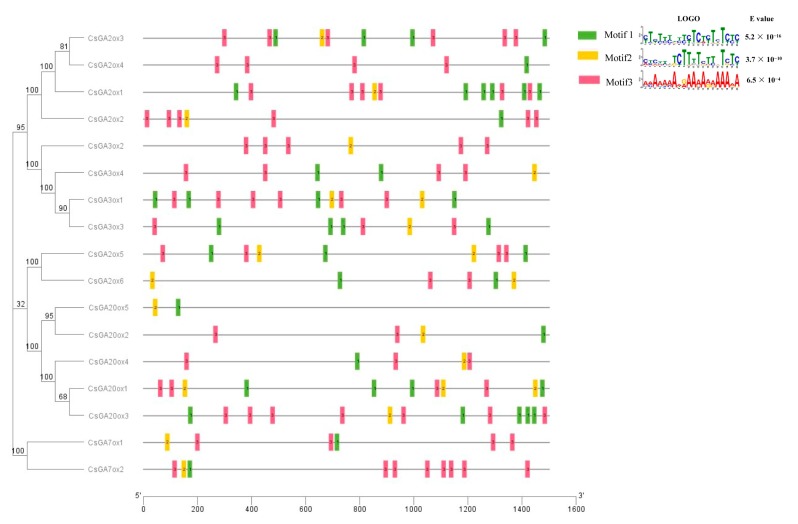
Putative conserved motifs in the promoters of *CsGAoxs* according to phylogenetic relationship. The 3 motifs were identified online using the MEME with 1.5 kb upstream region of start condon of all *CsGAox* genes. The following parameters “nmotifs 3, minw 6, maxw 20, minsites 30, maxsites 100” were used in MEME. Different motifs are indicated by different colors and are numbered 1–3. The logos of 3 conserved domain sequences, which were shown on the top right corner, were obtained from MEME Suite website. The bit score shows the information content of each position in the amino acid sequence.

To analyze conserved sequences potentially involved in the regulation of *CsGAox* genes, we selected a 1.5 kb upstream region from the start codon of each *CsGAox* gene. Three conserved motifs were identified in the promoters of all *CsGAoxs* by Multiple Em for Motif Elicitation (MEME) suite (Figure 3). To know if these motifs are potential transcription factor binding sites, the Regulation Prediction tool in PlantRegMap was used to scan transcription factor (TF) binding sites in the promoters of *CsGAoxs*. 18 TFs possess over-represented targets in the input gene set under cutoff *p* value ≤ 0.05 (Table 1). Among these TFs, 12 and 13 *CsGAox* genes were candidate target of BBR/BPC TF Csa2G365700 and GRAS TF Csa5G569350, respectively. Interestingly, the positions of BBR/BPC and GRAS binding sites were consistent with the positions of 3 conserved motifs which were identified by MEME (Table S2). Furthermore, we downloaded the BBR/BPC (Matrix_id MP00253 and MP00540) and GRAS (Matrix_id MP00611) TF binding motifs from PlantTFDB, and scanned these 3 binding motifs using FIMO. The 3 binding motifs were found in promoters of all 17 *CsGAox* genes (Figure 3, Table S3), indicating BBR/BPC and GRAS TFs may directly bind the 1 or more conserved motifs in the promoters of *CsGAoxs* to regulate their expression. 

### 2.6. Expression Patterns of CsGAox Genes in Response to GA3 and Uni Treatments in Roots of Cucumber Seedlings

To gain insights into the role of the *CsGAox* genes in cucumber growth and development, the expression of cucumber *GAox* genes in 10 tissues was analyzed regarding the published RNA-seq data [42]. Eleven *CsGAox* genes showed high levels of transcript abundance (FPKM (Fragments Per Kilobase Million) > 2.0) in at least 1 tissue (Figure S4). The transcripts of six genes (*CsGA2ox1*, *CsGA2ox3*, *CsGA2ox5*, *CsGA2ox6*, *CsGA20ox1* and *CsGA7ox1*) can be detected in all 10 tissues tested. Eight *CsGAox* genes (*CsGA2ox2*, *CsGA2ox3*, *CsGA2ox4*, *CsGA3ox2*, *CsGA7ox1*, *CsGA7ox2*, *CsGA20ox1* and *CsGA20ox3*) were highly expressed in roots (Figure S4), indicating they may play roles in root development.

**Figure 4:**
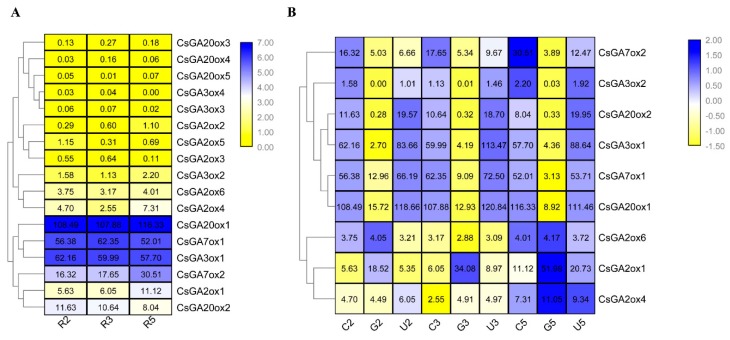
Heatmap of the expression profiles of *CsGAox* family genes in roots under control, GA_3_ and Uni treatments. (**A**) The developmental expression pattern analysis of *CsGAox* family genes at 3 developmental stages during roots growth. R2, R3 and R5 represent roots at 2, 3 and 5 days after seed germination under normal condition; Clustering was based on log2-transformed FPKM values of 17 *CsGAox* genes; (**B**) Expression pattern analysis of *CsGAox* family genes at 3 developmental stages of root under control, GA_3_ and Uni treatments. C2, C3 and C5 represent roots at 2, 3 and 5 days after seed germination under normal condition; G2, G3 and G5 represent roots at 2, 3 and 5 days after seed germination under 50 μM GA_3_ treatment; U2, U3 and U5 represent roots at 2, 3 and 5 days after seed germination under 10 μM Uni treatment. Clustering was based on Z-score row-scaled for each gene after log2-transformed FPKM values. The expression data was gained from the RNA-seq data with three biological replicates. Values, which were shown on the heatmaps, represent the average FPKM value of three biological replicates.

Based on above results, we performed RNA-seq deriving from the roots of cucumber seedlings at 2, 3 and 5 days after seed germination with 3 biological replicates. In our RNA-seq, the transcript abundance of 8 genes (*CsGA2ox2*, *CsGA2ox3*, *CsGA2ox5*, *CsGA3ox3*, *CsGA3ox4*, *CsGA20ox3*, *CsGA20ox4* and *CsGA20ox5*) was very low (FPKM < 2.0 in all 3 stages). The other 9 *CsGAox* genes (*CsGA2ox1*, *CsGA2ox4*, *CsGA2ox6*, *CsGA3ox1*, *CsGA3ox2*, *CsGA20ox1*, *CsGA20ox2*, *CsGA7ox1* and *CsGA7ox2*) showed high levels of transcript abundance (FPKM > 2.0) in 2 days, 3 days and 5 days roots (Figure 4A). It is worthy to note that *CsGA20ox1* showed highest expression in roots compared to other *CsGAox* genes (Figure 4A), which was consistent with the results of Figure S4. 

To analyze the expression profiles of *CsGAox* genes under GA_3_ and Uni treatments, RNA-seq of the roots of cucumber seedlings treated with GA_3_ and Uni for 2, 3 and 5 days after seed germination were performed. As only 9 *CsGAox* genes expressed well in roots in our RNA-seq data (Figure 4A), we further analyzed the expression changes of these 9 *CsGAox* genes (Figure 4B). As shown in Figure 4B, six *CsGAox* genes (*CsGA3ox1*, *CsGA3ox2*, *CsGA7ox1*, *CsGA7ox2*, *CsGA20ox1* and *CsGA20ox2*) were strongly repressed by GA_3_ treatments in all 3 stages, especially for *CsGA20ox1*. Among these 6 genes, *CsGA3ox1* and *CsGA20ox2* were significantly induced by Uni treatment in all 3 stages. However, *CsGA7ox2* were repressed by both GA_3_ and Uni treatments in all 3 stages. In contrast, three *GA2ox* genes (*CsGA2ox1*, *CsGA2ox4* and *CsGA2ox6*,) were simultaneously induced by GA_3_ treatment under at least one stages, but not Uni treatment (Figure 4B). 

### 2.7. Overexpressing CsGA20ox1 in Arabidopsis Promotes Primary and Lateral Root Development

**Figure 5:**
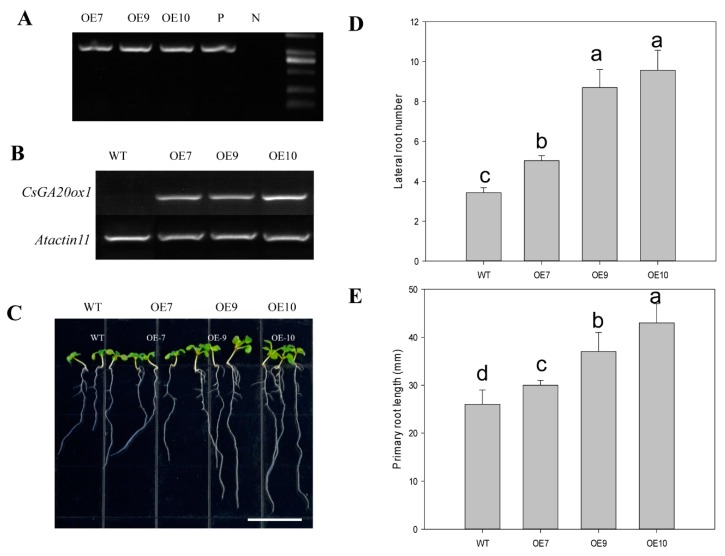
Ectopic expression of *CsGA20ox1* in *Arabidopsis*. (**A**) *Arabidopsis* transformants were confirmed by Polymerase chain reaction (PCR) using CaMV35S promoter specific forward primer together with *CsGA20ox1* reverse primer; (**B**) Relative expression of *CsGA20ox1* in Col-0 and three T3 generation transgenic lines. Total RNA was extracted from 10-day-old seedlings, then analyzed by semi-quantitative RT-PCR. The *AtActin11* gene was used as an internal control; (**C**) The morphology of 10-day-old seedlings of Col-0 and three over-expression (OE) lines; (**D**,**E**) Lateral root number (**D**) and the primary root length (**E**) in 10-day-old wild type (Col-0) and three OE lines. Values are the means with SE (*n* = 8–10). Different letters indicate significant differences between means (ANOVA with Tukey’s HSD test, *p* < 0.05).

As *CsGA20ox1* is highly expressed in roots and the expression was mostly repressed by GA_3_ treatments in our study, *CsGA20ox1* was further ectopically expressed in *Arabidopsis* to study whether *CsGA20ox1* involves in root development. Homozygous T3 lines of 3 independent T0 lines with different expression levels of *CsGA20ox1* were selected to perform phenotypic analysis (Figure 5A,B). *CsGA20ox1-OE* lines grown on vertical MS-agar plates for 10 days produced significantly longer primary roots (Figure 5C) than wild type. The results also showed that the lateral root number of primary root of CsGA20ox1-OE lines 7, 9, and 10 increased by 46.7%, 153.6%, and 178.9%, respectively, compared to that of wild type (Figure 5C–E). These results demonstrated that overexpression of *CsGA20ox1* in *Arabidopsis* promotes primary and lateral root development.

## 3. Discussion

GAs are plant hormones that are essential for many developmental processes in plants. GA biosynthesis is complex, the final part of the pathway is catalysed by GAox, including GA20ox, GA3ox, and GA2ox in most plants [38,43]. One additional enzyme, GA7 oxidase (GA7ox), which catalyzes the C-7 oxidation of GA12 aldehyde to form GA12 [37], was only identified in specific plant lineages (e.g., Cucurbits) [18,44]. In this study, 5 *GA20oxs*, 4 *GA3oxs*, 6 *GA2oxs* and 2 *GA7oxs* were identified in cucumber. Compared to rice, the number of *GA20oxs* and *GA2oxs* were less, but the number of *GA3oxs* was bigger in cucumber and *Arabidopsis*, which suggested that the *GA3ox* subfamily in cucumber and *Arabidopsis* had expanded compared to rice, but not *GA20ox* and *GA2ox* subfamilies. Gene duplication events were the most important for the rapid expansion and evolution of gene families [41] and 1 segmental duplication event (*CsGA3ox1* and *CsGA3ox2*) was identified in cucumber using MCScanX (Figure 2A, Table S1), indicating that segmental duplication served as a driving force throughout *GA3ox* evolution and this may be one of the possible reasons that the *GA3ox* subfamily in cucumber had expanded compared to rice. In addition, 1 segmental duplication event (*CsGA2ox3* and *CsGA2ox4*) and 1 tandem duplication event (*CsGA7ox1* and *CsGA7ox2*) was identified in *CsGA2ox* and *CsGA7ox* subfamily, respectively. Duplication event was not found in *GA20ox* subfamily in cucumber. 

To obtain an overall picture of the 17 cucumber GAox proteins and their relationships with those of *Arabidopsis* and rice, a phylogenetic trees combining cucumber, *Arabidopsis* and rice GAox proteins was constructed, which divided the 52 GAox into 5 subfamilies. The 17 CsGAox members fell into all 5 subfamilies. Interestingly, GA7ox subfamily only includes 2 CsGA7oxs and 2 OsGAoxs, but did not include any *Arabidopsis* GAox, which suggested that this subfamily was either lost in *Arabidopsis* or was acquired in cucumber and rice after divergence from the last common ancestor. It is worthy to note that no GA7ox was identified in rice in the previous study and the 2 OsGAoxs, previously named OsGA20ox5 and OsGA20ox8 [20], were clustered in GA7ox subfamily in this study. Han and Zhu (2011) also demonstrated that OsGA20ox5 and OsGA20ox8 were outside the four subfamilies (GA20ox, GA3ox, C19 GA2ox, and C20 GA2ox) in phylogenetic trees combining rice, *Arabidopsis* and soybean GAox proteins [20]. These results indicated that OsGA20ox5 and OsGA20ox8 are homologs of CsGA7ox1 and CsGA7ox2 in rice. OsGA20ox8 was the closest homolog of CsGA7ox1 in rice, indicating that it may have similar function with CsGA7ox1, which is a multi-functional enzyme with 7-oxidase,3β- and 15 α-hydroxylase activity [36]. However, their biological functions remain to be studied in the future. 

To further study the evolutionary relationship and diversity/conservativeness of *GAox* genes in cucumber, *Arabidopsis* and rice, the gene structure, domain and motif were analyzed. All of the *GAox* genes in cucumber, *Arabidopsis* and rice had both 2OG-FeII_Oxy and DIOX_N domains, which are characteristics of GAox proteins. The pattern of gene structural diversity and motif composition can provide important evidence for evolutionary relationships of multi-gene families [45,46]. Comparing the phylogenetic tree with the gene structure and motif analysis, it is apparent that the most closely related members within subfamilies shared similar gene structure and motif composition (Figure 1). In addition, comparative syntenic map analysis between cucumber, *Arabidopsis* and rice genomes was performed to explore the origin and evolutionary process of cucumber *GAox* genes. The *GAox* genes were located in syntenic regions also showing highly conserved gene structure and motif composition (Figure 1 and Figure 2). These results indicated that similar functions among members of the same *GAox* subfamily and genes located in syntenic regions and the similar selection acting on them. 

The shallow root system of cucumber is one of the most severe limitations in cucumber production, such as water and nutrient uptake, biotic and abiotic stress tolerance [47,48]. Increasing evidence showed that GA regulates root development, especially lateral root formation [49,50,51]. However, GA plays different roles in regulating lateral root formation in different plants. For example, GAs negatively affected lateral root formation in *Populus* [49] and rice [23]. However, shoot-applied GA showed some promoting effects on lateral root formation in *Arabidopsis* [52]. As GA oxidases are critical enzymes in GA synthesis pathway, it is important to study how *GAox* genes response GA_3_ and Uni treatments and what the functions of *GAoxs* are in cucumber root development. Previous RNA-seq data indicated that 8 *CsGAoxs* highly expressed in roots (Figure S4) [42], indicating these CsGAoxs may play important roles in root development. Therefore, RNA-seq data of roots under 50 μM GA3 and 10 μM Uni treatments was used to analyze the expression pattern of *CsGAoxs*. In our study, 9 *CsGAoxs* expressed well in roots and the expression of all of 9 *CsGAoxs* was regulated by GA_3_ and only 3 genes (*CsGA3ox1*, *CsGA20ox2* and *CsGA7ox2*) can responds to both GA_3_ and Uni treatments. In addition, 5 out of 8 genes were significantly repressed by GA_3_ treatments at all 3 stages and three *GA2ox* genes (*CsGA2ox1*, *CsGA2ox4* and *CsGA2ox6*,) were simultaneously induced by GA_3_ treatment under at least one stage (Figure 4). 

In addition, *CsGA20ox1* showed the highest expression in root among the *CsGAoxs*, which was consistent with the previous study (Figure S4) [42]. The final steps to produce active GAs require activity of the GA20ox and the level of endogenous active GA is governed by feedback regulation [14,53,54]. In our study, we also found that the expression of *CsGA20ox1* was strongly repressed by GA_3_ (Figure 4), indicating that *CsGA20ox1* maybe an important gene in roots for GA response and synthesis. To further study the function of *CsGA20ox1*, we generated transgenic *Arabidopsis* that overexpressed *CsGA20ox1*. The transgenic lines showed longer primary root and more lateral roots than the WT seedlings. These results indicated that over expression of *CsGA20ox1* promotes primary root elongation and lateral root formation (Figure 5). One possible reason is that over expression *CsGA20ox1* results in increased GA concentration in transgenic lines, which is consistent with exgenous GA application promotes primary and lateral roots formation in *Arabidopsis* [52]. 

In this study, CsGA20ox1 was grouped together with AtGA20ox1, AtGA20ox2, AtGA20ox3 and AtGA20ox4 (Figure 1A). It has been proposed that *AtGA20oxs* control various aspects of plant development, especially growth of most vegetative tissues, floral transition, floral organ growth and anther development [54]. However, little is known about the function of *AtGA20oxs* in regulating root development, indicating that CsGA20ox1 and AtGA20oxs may have functional divergence. Moreover, BBR/BPC and GRAS were identified as candidate TFs which can directly bind the promoters of *GAoxs*. The BBR/BPC family is a poorly characterized plant transcription factor family of GAGA BINDING PROTEINS [55,56]. GRAS TFs are major players in GA signaling and some GRAS TFs were demonstrated to be involved in root development [57,58]. However, the function and relationship of BBR/BPC, GRAS and *CsGA20ox1* should be further studied in the future.

## 4. Materials and Methods

### 4.1. Identification of GAox Genes in Cucumber

Sixteen *Arabidopsis* GAox proteins were used as query sequences and Blastp searches against the predicted cucumber proteins. In addition, the Hidden Markov Model (HMM) profile of 2OG-FeII_Oxy (PF03171) and DIOX_N (PF14226) from the Pfam database (available online: http://pfam.janelia.org) was also applied as a query to search the *GAox* genes from the cucumber genome database. All candidate genes were further examined by confirming the existence of both 2OG-FeII_Oxy (PF03171) and DIOX_N (PF14226) domains using the Pfam and Simple Modular Architecture Research Tool (SMART) program.

Proteins of GAoxs in *Arabidopsis* and rice were downloaded from The Arabidopsis Information Resource (TAIR) database (available online: https://www.arabidopsis.org) and the Rice Genome Annotation Project Database (available online: http://rice.plantbiology.msu.edu/), which was described in previous reports [14,20].

### 4.2. Phylogenetic Analysis

Multiple sequence alignments were performed using ClustalX 1.81 with default parameters and DNAMAN (available online: https://www.lynnon.com/). The phylogenetic trees were constructed with the full protein sequences of 55 GAoxs using MEGA 7.0 (available online: https://www.megasoftware.net/) [59]. The neighbor-joining (NJ) method was used with the following parameters: Poisson correction, pairwise deletion, and bootstrap (1000 replicates; random seed).

### 4.3. Gene Structure Analysis, Conserved Motif Recognition and Transcription Binding Site Analysis

The DNA and cDNA sequences corresponding to each predicted gene from cucumber genome (Chinese Long 9930) were downloaded, and the gene structures were analyzed using the web-based bioinformatics tool GSDS (available online: http://gsds.cbi.pku.edu.cn/) [60]. MEME (Multiple Expectation Maximization for Motif Elicitation) was used to identify conserved motif structures of GAox protein and promoter sequences [61,62]. 

The transcription binding sites were analyzed at Plant Transcriptional Regulatory Map (PlantRegMap, available online: http://plantregmap.cbi.pku.edu.cn/regulation_prediction.php). The transcription binding motifs were downloaded at Plant Transcription Factor Database (PlantDFTB, available online: http://planttfdb.cbi.pku.edu.cn/download.php) [63]. Motif scanning were performed by Find Individual Motif Occurences (FIMO, available online: http://meme-suite.org/tools/fimo) [64]. 

### 4.4. Chromosomal Distribution and Gene Duplication

All *CsGAox* genes were mapped to cucumber chromosomes based on physical location information from the database of cucumber genome using Circos [65]. Multiple Collinearity Scan toolkit (MCScanX) was adopted to analyze the gene duplication events, with the default parameters [66]. To exhibit the synteny relationship of the orthologous *GAox* genes obtained from cucumber, *Arabidopsis* and rice, the syntenic analysis maps were constructed using TBtools (available online: https://github.com/CJ-Chen/TBtools) [67]. 

### 4.5. Plant Materials, Treatment, Sample Collection, RNA Extraction and RNA-Seq

A north China-type cucumber cultivar “Daqingba” was used in this study for GA_3_ and Uni treatments and RNA-seq. The seeds of “Daqingba” were cultured in MS medium in 9 cm petri dish. After germination, the seeds with similar germination status were transferred to MS medium supplied with 50 μM GA_3_ and 10 μM Uni in glass jar. The seeds were also transferred to MS medium in glass jar without any treatment as control. Each jar contained 10 seeds, with 2 jars per biological replicate and three biological replicates for each treatment. All cultures were maintained in a culture room at 25 ± 1 °C with a 16 h photoperiod and a light intensity of 80 μmol m^−2^ s^−1^ provided by cool-white fluorescent lights.

The roots were harvested on the 2nd, 3rd and 5th day after treatments. 0.5 cm distal end of primary, secondary and tertiary roots and 0.5 cm proximal end of secondary and tertiary roots were collected, and if the secondary and tertiary roots were not longer than 1.0 cm, the whole roots were collected. Total RNA was extracted from a mixture of the root tissues of 10–20 individual plants as one replication for each treatment. Total RNAs were extracted from the samples using TRIzol reagent (Invitrogen, Carlsbad, CA, USA) and treated with DNase I (Fermentas, Burlington, ON, Canada) according to the manufacturers’ instructions. The integrity of the RNA was verified through RNase-free agarose gel electrophoresis, and the concentration was measured using RNA Nano6000 Assay Kit of the Bioanalyzer 2100 system (Agilent Technologies, Santa Clara, CA, USA). The cDNA library preparation and sequencing were conducted by the Allwegene Technology Company in Beijing, China. All libraries were sequenced on the Illumina HiSeq 4000TM platform (Illumina, Inc., San Diego, CA, USA). Gene expression levels were analyzed by employing the fragments per kilobase of exon model per million mapped reads (FPKM) algorithm, a commonly used method to measure the level of gene expression. The heatmaps were generated using TBtools (available online: https://github.com/CJ-Chen/TBtools) [67].

### 4.6. Overexpression Vector Construction, Arabidopsis Transformation, Gene Expression and Phenotypic Analysis

The full-length coding sequence (CDS) of *CsGA20ox1* was PCR amplified with primers 5′-GCGGATCCCCTCCCCATGGCTTTTCTTT-3′ and 5′-GCGAGCTCCTAAGGAAAGAAGAGAGGAAG-3′ and inserted into the expression vector pBI121 between the BamHI and SacI sites. The resultant plasmid (pBI121-CaMV35S::CsGA20ox1) was transformed into the *Agrobacterium tumefaciens* strain LBA4404 using the freeze–thaw method, which was used for transformation of *Arabidopsis* plants (Col-0) by the floral dip method [68]. Homozygous T3 transgenic *Arabidopsis* lines were identified by kanamycin (50 mg/L) selection and PCR with 35S primer 5′-GTATGGACGATTCAAGGC-3′ and *CsGA20ox1* primer 5′-CTGGCATAACCACAATGTTCG-3′. Semi-quantitative RT-PCR assay was performed with *CsGA20ox1* primers 5′-GCATAGAGCAGTGGTGAA-3′ and 5′-ATTGGAGAAGCATGGACC-3′. The *Arabidopsis Actin11* was used as an internal control in semi-quantitative RT-PCR assay (5’-CCACATGCTATTCTGCGTTTGGACC-3′ and 5′-CATCCCTTACGATTTCACGCTCTGC-3′). For the root length assay, seeds were germinated and grown vertically on MS agar medium for 10 days, after which root length was measured with a ruler and photographed. 

## Figures and Tables

**Table 1:** Candidate transcription factors binding promoters of *CsGAoxs* identified by PlantRegMap.

Transcription Factor	Family	Query All	Query Bind	*p* Value	*q* Value
Csa3G686200.1	MADS-box	17	4	1.64 × 10^−3^	1.13 × 10^−1^
Csa6G499720.1	HD-ZIP	17	3	2.21 × 10^−3^	1.13 × 10^−1^
Csa2G365700.1	BBR-BPC	17	12	4.59 × 10^−3^	1.27 × 10^−1^
Csa5G569350.1	GRAS	17	13	5.69 × 10^−3^	1.27 × 10^−1^
Csa6G502050.1	C2H2	17	4	6.22 × 10^−3^	1.27 × 10^−1^
Csa5G641610.1	MYB	17	2	1.04 × 10^−2^	1.55 × 10^−1^
Csa3G122500.1	MYB	17	4	1.12 × 10^−2^	1.55 × 10^−1^
Csa3G826680.1	ZF-HD	17	2	1.39 × 10^−2^	1.55 × 10^−1^
Csa1G029620.1	C2H2	17	4	1.55 × 10^−2^	1.55 × 10^−1^
Csa5G270900.1	C2H2	17	3	1.71 × 10^−2^	1.55 × 10^−1^
Csa3G165680.1	MYB	17	2	2.00 × 10^−2^	1.55 × 10^−1^
Csa6G136580.1	MYB	17	5	2.13 × 10^−2^	1.55 × 10^−1^
Csa6G502710.1	ZF-HD	17	2	2.94 × 10^−2^	1.77 × 10^−1^
Csa2G270220.1	MYB	17	3	2.96 × 10^−2^	1.77 × 10^−1^
Csa3G848250.2	C2H2	17	3	3.20 × 10^−2^	1.77 × 10^−1^
Csa4G054800.1	Nin-like	17	2	3.48 × 10^−2^	1.77 × 10^−1^
Csa1G033200.1	MYB	17	4	4.38 × 10^−2^	1.93 × 10^−1^
Csa2G301510.1	Trihelix	17	2	4.93 × 10^−2^	2.01 × 10^−1^

## Supplementary Materials

Supplementary materials can be found at http://www.mdpi.com/1422-0067/19/10/3135/s1.
