# Supplementary material for: Comprehensive Analysis of Cucumber Gibberellin Oxidase Family Genes and Functional Characterization of CsGA20ox1 in Root Development in Arabidopsis
Source: Int J Mol Sci. 2018 Oct 12;19(10):3135. doi: 10.3390/ijms19103135 (PMC6213227; doi:10.3390/ijms19103135)
Supplement: Supplementary file 1 [file ijms-19-03135-s001.zip › Supplementary figures.pdf]

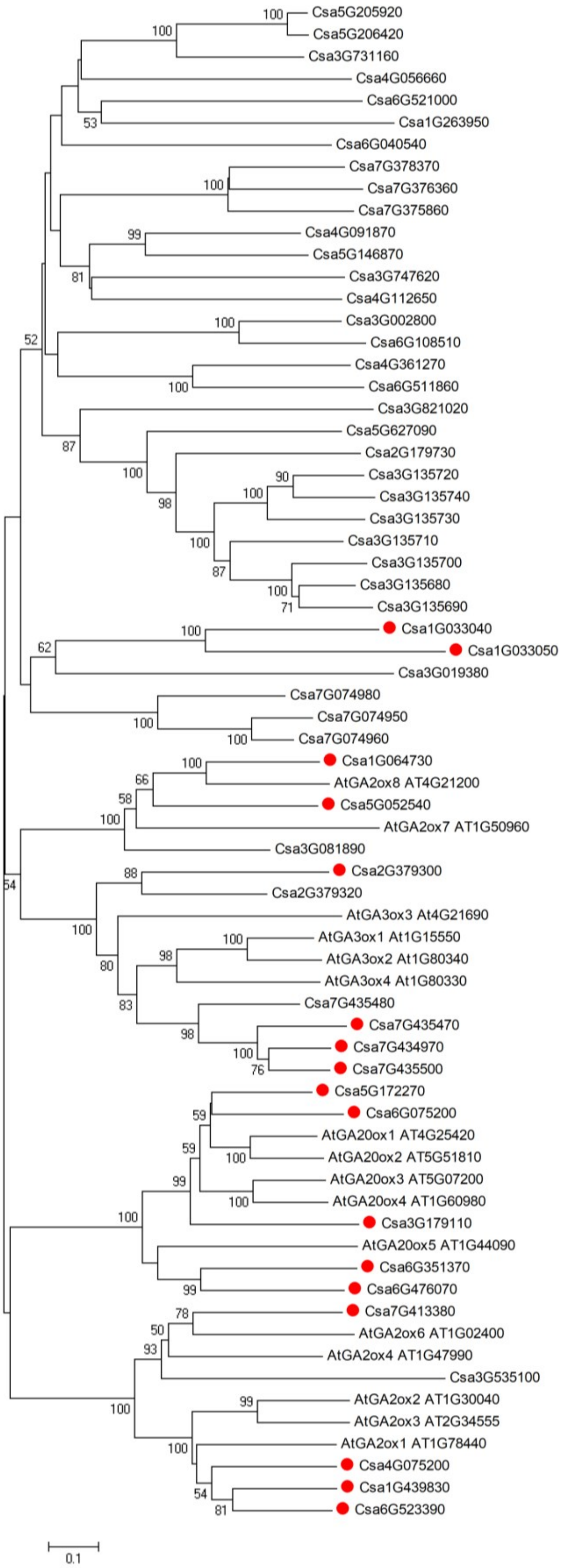

Figure S1. Phylogenetic analysis candidate *GAox* genes in cucumber. The neighborjoining tree includes 53 candidate GAox proteins from cucumber and 16 AtGAox proteins. The 17 CsGAox were labeled by red dots.

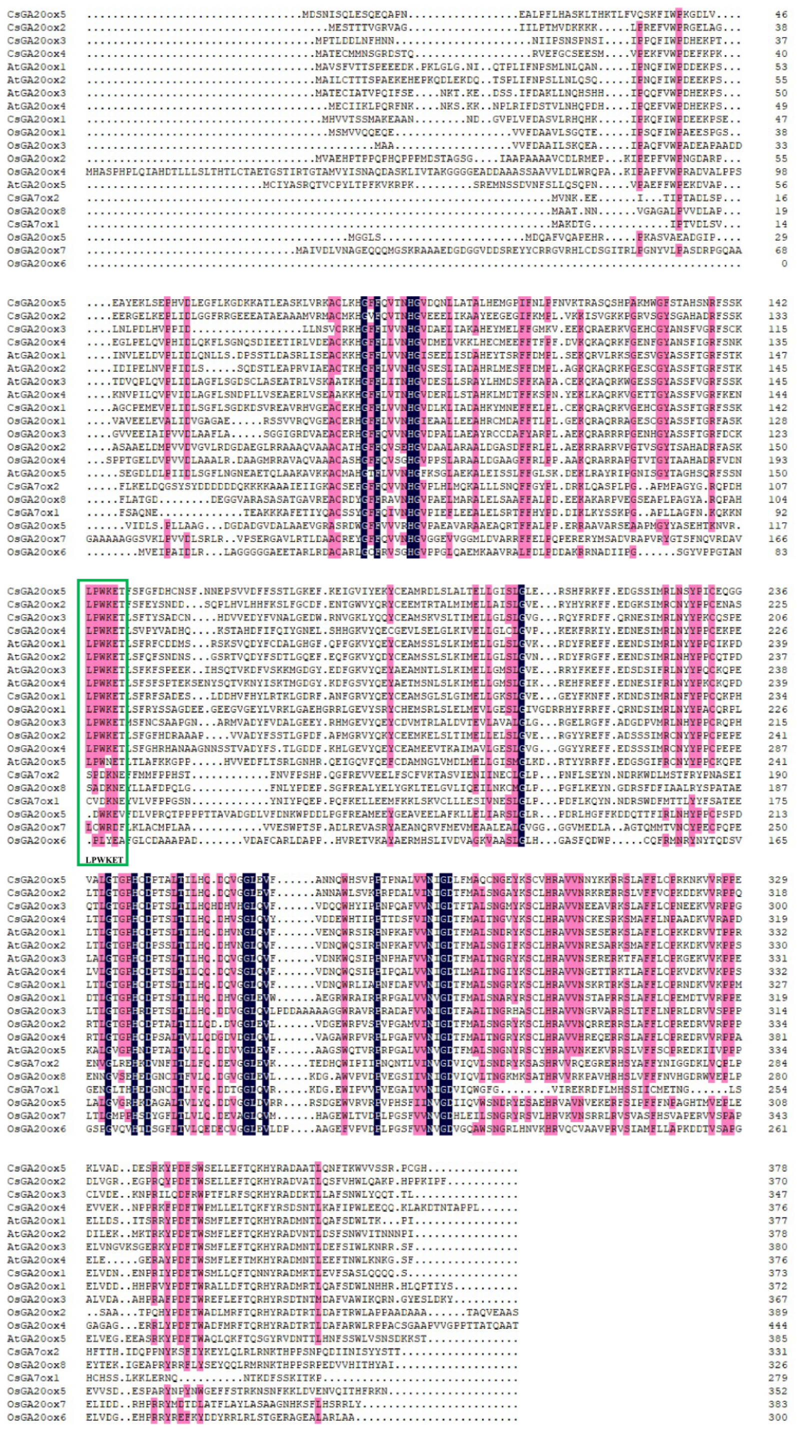

Figure S2. Multiple sequence alignments of GA20ox proteins from 5 CsGA20ox proteins, 5 AtGA20ox proteins, 8 OsGA20ox protein and 2 CsGA7ox proteins. The GA20ox conserved sequence LPWKET was labeled in green box. Amino acid identity of 100% is marked with a black background, amino acids identity of higher than 75% is marked with a pink background, amino acids identity of more than 50% is marked with a light green background.

	Logo	E-value	Sites	Width
1.	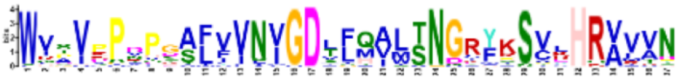	3.5e-1078	51	37
2.	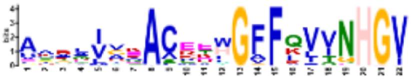	2.5e-537	52	22
3.	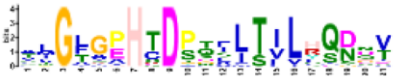	2.3e-490	52	21
4.	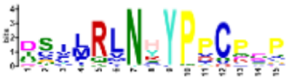	2.9e-310	49	15
5.	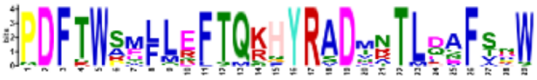	1.9e-221	14	29
6.	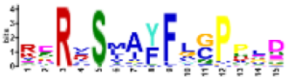	1.5e-242	51	15
7.	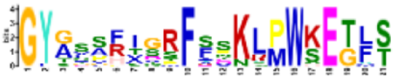	4.8e-216	23	21
8.	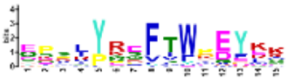	9.1e-179	49	15
9.	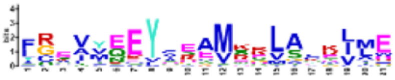	2.3e-260	48	21
10.	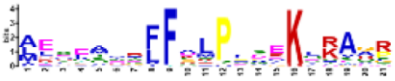	5.6e-205	51	21

Figure S3. Ten conserved motifs from all GAox proteins from cucumber, *Arabidopsis* and rice. The logos of ten conserved motif sequences were obtained from MEME Suite website. The bit score means information content of each position in the amino acid sequence.

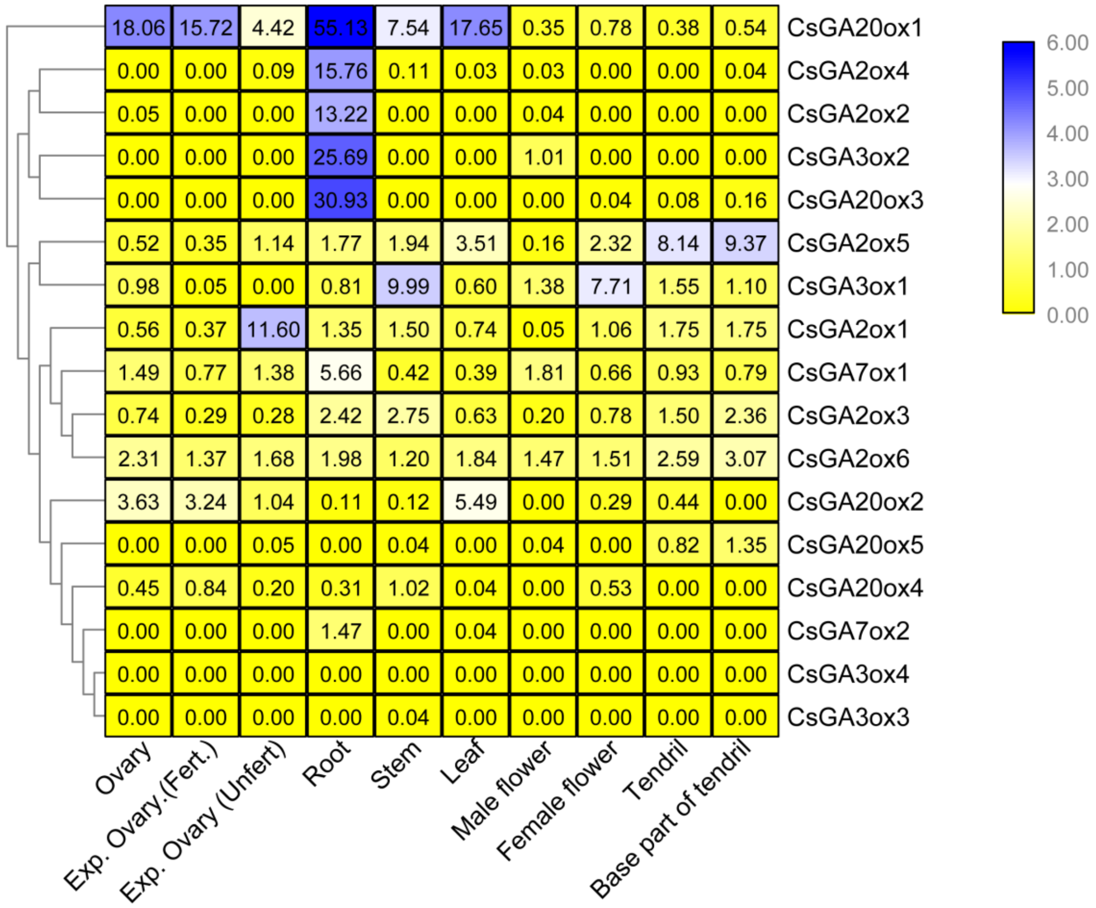

Figure S4. Heatmap of the expression profiles of *CsGAox* family genes in 10 cucumber tissues. Values, which were shown on the heatmap, represent the average FPKM value of three biological replicates. Clustering was based on log2-transformed FPKM values of 17 *CsGAox* genes. Ovary, cucumber ovary tissue (unexpanded); Exp. Ovary.(Fert.), cucumber expanded ovary tissue (fertilized); Exp. Ovary.(UnFert.), cucumber expanded ovary tissue (unfertilized).
